# Immune mediators as plasma biomarkers for identifying household contacts and classifying clinical forms and leprosy reactions

**DOI:** 10.3389/fimmu.2025.1513060

**Published:** 2025-03-05

**Authors:** Jairo Campos Carvalho, Marcelo Antônio Pascoal-Xavier, Marcelo Grossi Araújo, Júlia Pereira Martins, Andrea Teixeira-Carvalho, Matheus de Souza Gomes, Laurence Rodrigues Amaral, Vanessa Peruhype-Magalhães, Jordana Grazziela Alves Coelho-dos-Reis, Olindo Assis Martins-Filho, Márcio Sobreira Silva Araújo

**Affiliations:** ^1^ Instituto René Rachou/FIOCRUZ, Grupo Integrado de Pesquisas em Biomarcadores, Belo Horizonte, Minas Gerais, Brazil; ^2^ Fundação Hospitalar do Estado de Minas Gerais (FHEMIG), Belo Horizonte, Minas Gerais, Brazil; ^3^ Universidade Federal de Minas Gerais, Faculdade de Medicina, Departamento de Anatomia Patológica e Medicina Legal, Belo Horizonte, Minas Gerais, Brazil; ^4^ Universidade Federal de Minas Gerais, Serviço de Dermatologia do Hospital das Clínicas, Belo Horizonte, Minas Gerais, Brazil; ^5^ Laboratório de Bioinformática e Análises Moleculares, Universidade Federal de Uberlândia, Uberlândia, MG, Brazil; ^6^ Universidade Federal de Minas Gerais, Instituto de Ciências Biológicas, Departamento de Microbiologia, Laboratório de Virologia Básica e Aplicada, Belo Horizonte, Minas Gerais, Brazil

**Keywords:** leprosy, cytokine, chemokines, Luminex, decision tree algorithm

## Abstract

The present study aimed to evaluate the performance of plasma immune mediators in classifying leprosy patients [L(PB) and L(MB), paucibacillary and multibacillary leprosy, respectively], leprosy reaction patients (T1LR and T2LR, type 1 and type 2 leprosy reaction, respectively), household contacts (HHC), and non-infected (NI) controls. Quantitative measurements of these immune mediators were carried out using high-throughput multiplex microbead array. The results demonstrated that most of the plasma immune mediators were increased in all clinical groups compared with NI controls. Higher frequencies but lower maximum magnitudes of increase (fold change according to NI) were observed for T1LR (63%, 6.1×) and T2LR (63%, 9.7×) compared with HHC (48%, 68.5×), L(PB) (56%, 8.5×), and L(MB) (48%, 37.9×). The bi-dimensional scattering profiles (magnitude order *vs*. significance) identified a higher number of immune mediators in T2LR (12/27) compared with HHC (8/27), L(PB) (7/27), L(MB) (5/27), and T1LR (5/27). CXCL8 was selected as the parameter with the highest accuracy and significance [area under the receiver operating characteristic curve (AUC) = 0.98, *p* = 0.0002] in classifying NI *vs*. HHC. CCL3 (C–C motif chemokine ligand 3) was the single analyte with moderate accuracy and significance (AUC = 0.74, *p* = 0.0422) in classifying L(PB) *vs.* L(MB). IL-9 was selected as an attribute with moderate accuracy and significance (AUC = 0.77, *p* = 0.0041) in classifying T1LR *vs.* T2LR. Decision tree algorithms confirmed the high accuracy (96%) of CXCL8 in classifying NI *vs*. HHC. The use of CCL3 followed by IFN-γ classified L(MB) *vs.* L(PB) with high accuracy (93%). Moreover, the analysis of IL-9 followed by IL-6 and CXCL10 classified T1RL *vs.* T2RL with high accuracy (96%). In general, combined stepwise algorithms showed enhanced classification accuracy compared with single-attribute analysis. Together, our findings supported the potential use of plasma immune mediators as complementary laboratory biomarkers for the identification of HHC and the classification of distinct clinical forms of leprosy and leprosy reactions.

## Introduction

Leprosy is a highly neglected chronic contagious disease caused by *Mycobacterium leprae* and *Mycobacterium lepromatosis*, obligatory intracellular bacilli that affect the skin and nerves and can cause serious, potentially disabling damage. Despite the long history of this disease, leprosy remains a great threat to public health in developing countries. It is a disease with high detection rates worldwide, which affects people of all ages and can compromise their professional and social development. It is relevant to emphasize that leprosy is primarily a neural disease, with the bacillus infecting the peripheral nerves and leading to their thickening, particularly in the limbs, which can cause physical disability (1–3). Early diagnosis and timely treatment can prevent complications such as physical deformities and even disabling and permanent injuries (4). Leprosy is a disease that presents itself in a spectral form, characterized by clinical and immunopathological manifestations that express a direct relationship with the inflammatory mediators present during the disease progression and the development of specific clinical forms (5, 6). Patients in the paucibacillary group are those with the tuberculoid and borderline tuberculoid clinical forms who show an immune cellular response mediated mainly by macrophages, which will attempt to eliminate the bacillus during the course of the disease. Pro-inflammatory cytokines such as interferon gamma (IFN-γ), tumor necrosis factor (TNF), and interleukin 1β (IL-1β) are predominant in the lesions (7–12). The multibacillary group encompasses patients from the borderline–borderline and borderline–lepromatous groups and the lepromatous form of leprosy. These patients have a modulated immune response characterized by the production of the cytokines IL-4, IL-5, IL-10, and transforming growth factor beta (TGF-β) that favor bacillary dissemination (9, 11, 13, 14).

There are studies in the literature that associate the expression of circulating chemokines, cytokines, and growth factors with the clinical forms of leprosy. Others have even mentioned their possibility of serving as biomarkers for prognosis and diagnosis; however, several of these are inconclusive (6, 15–20). Determining the profiles of plasma soluble biomarkers capable of identifying asymptomatic individuals, i.e., those infected with *M. leprae*, but without the clinical symptoms of leprosy, can be an important tool to reduce bacillus transmission and to prevent deficiencies (11, 21–25).

Therefore, considering the importance of laboratory research tools in aiding the clinical management of patients with leprosy, the present study aimed to identify circulating immunological biomarkers from plasma that could contribute as predictors of the progression of disease and the development of infection in household contacts. For this, statistical tools such as decision tree (DT) algorithms methods and the receiver operating characteristic (ROC) curve were applied to evaluate the concentrations of 27 plasma immune mediators. The results indicate high performance of the selected biomarkers that comprised an immunological algorithm conspicuously differentiating the clinical forms of leprosy.

## Materials and methods

### Study population

This study comprised an observational study that enrolled patients with leprosy living in different municipalities in Minas Gerais State, Brazil. A total of 75 participants were enrolled as a non-probabilistic convenience sample. The study population comprised a total of 50 patients with leprosy, seven household contacts (HHC) of leprosy patients, and 18 non-infected (NI) healthy subjects. Patients with leprosy were invited to participate in the study at the Outpatient Dermatology Clinic of the Clinics Hospital of Universidade Federal de Minas Gerais. The inclusion criteria for patients with leprosy were: volunteers of both sexes; older than 18 years with no previous selection based on ethnic features; and educational level or socioeconomic class who agreed to participate in the study. The patients had no history of multidrug therapy and no records of immunosuppressive treatment. Patients with leprosy were categorized according to the operational classification into subgroups. The paucibacillary leprosy [L(PB)] group L(PB);(*n* = 14; six men and eight women, with a median age of 38 years) included those patients with up to five cutaneous lesions, one compromised nerve, and a negative bacteriological index (BI) in the slit-skin smears. The multibacillary leprosy [L(MB)] group L(MB); (*n* = 13; seven men and six women, with a median age of 48 years) comprised patients who had more than five cutaneous lesions, two or more affected nerves, and/or a positive BI. Patients with leprosy presenting reversal reactions were included in the subgroup referred to as type 1 leprosy reaction (T1LR) (*n* = 12, nine men and three women, with a median age of 49 years). The T1LR group included patients who presented with sudden erythematous plaques over previous skin lesions and new lesions with the same morphology, in addition to acute neuritis. Patients with clinical signs of erythema nodosum leprosum comprised the type 2 leprosy reaction (T2LR) subgroup (*n* = 11, 10 men and one woman, with median age of 45 years). Patients in the T2LR group were those with the multibacillary form of the disease who developed lesions of erythema nodosum leprosum. The HHC group (*n* = 7, two men and five women, with a median age of 38 years) was composed of healthy volunteers living in endemic areas for leprosy who are companions of leprosy patients, with no clinical signs of leprosy, who were contacted at the outpatient unit. All HHC were submitted to clinical evaluation and did not present any neurological and/or dermatological signs of leprosy. A group of NI healthy individuals served as the control group (*n* = 18, eight men and 10 women, with a median age of 38 years), which was composed of volunteers with no family and personal clinical history of leprosy and living in the metropolitan area of Belo Horizonte, Minas Gerais, Brazil. All were interviewed by an infectious disease specialist and did not report any clinical signs. **Table 1** summarizes the major demographic and clinical features of the study population.

**Table 1:** Demographic, laboratorial and clinical features of the study population.

Groups	(n)	Sex		AgeMedian (Min-Max)	Bacilloscopic IndexMedian (Min-Max)	Number of LesionsMedian (Min-Max)	Affected Nerves Median (Min-Max)
		Malen (%)	Femalen (%)				
NI	18	8 (44.4)	10 (55.6)	39 (23-56)	-	-	-
HHC	7	2 (28.6)	5 (71.4)	38 (21-54)	–	–	–
L(PB)	14	6 (42.9)	8 (57.1)	39 (31-47)	0 (0-0)	1.0 (1-5)	0 (0-3)
L(MB)	13	7 (53.8)	6 (46.2)	48 (34-58)	4.0 (2.5-5.5)*	20 (7-20)*	1.0 (0-3)
T1LR	12	9 (75.0)	3 (25)	49 (21-62)	0 (0-3.5)	20 (1-30)	2 (0-3)
T2LR	11	10 (90.9)	1 (9.1)	45 (27-64)	3.0 (2.8-5.0)**	20 (20-30)	2 (0-2)

NI = Non-Infected healthy volunteers with no clinical history of leprosy; HHC = households contacts of Leprosy patients; L(PB) = Paucibacillary Leprosy; MB – Multibacillary Leprosy patients; T1LR – patients with Type 1 Leprosy Reaction; T2LR – patients with Type 2 Leprosy Reaction. No significant difference was observed for age distribution by Kruskal-Wallis (p= 0.0714). No significant difference was observed for sex distribution by Chi-square Test (p=0.0633). *Higher bacilloscopic index and number of lesions were observed in L(MB) as compared with L(PB) by Mann-Whitney test (p= 0,0002; p= 0,0008, respectively); **Higher bacilloscopic index was observed in T2LR as compared to T1LR by Mann-Whitney test (p= 0,0242).

This study was submitted and approved by the Ethics Committee of Instituto René Rachou – FIOCRUZ-MG (Research Protocol CAAE: 77737317.1.0000.5149). All participants have read and signed the free and informed consent before inclusion in the study.

### Biological sample collection, processing, and storage

Whole blood specimens (10 ml) were collected from volunteers by venipuncture using vacuum tubes containing sodium heparin as an anticoagulant. Plasma samples were obtained by centrifugation at 1,400 × *g*, 4°C, for 10 min. The plasma aliquots were stored at −80°C until use to determine the levels of immune mediators using a high-throughput Luminex Bio-Plex microbead immunoassay.

### High-throughput Luminex Bio-Plex microbead immunoassay

Quantitative measurement of the plasma immune mediators was carried out with the “Bio-Plex Pro Human Cytokine 27-Plex” platform (Bio-Rad Laboratories, Hercules, CA, USA) according to the manufacturer’s instructions. Samples were run on a Bio-Plex 200 instrument using the Manager software, version 6.0 (Bio-Rad Laboratories, Hercules, CA, USA). A minimum of 50 beads were acquired for each analyte. The final concentration of plasma immune mediators was obtained according to standard curves using a five-parameter logistic fit regression to convert the mean fluorescence intensities into picograms per milliliter.

### Statistical analysis

Comparative analysis of the demographic, laboratory, and clinical features was carried out using chi-square, Kruskal–Wallis for multiple comparisons, and Mann–Whitney for two-group analysis according to the categorical or continuous distribution of each variable. Multiple comparative analyses of the concentrations of the plasma immune mediators among groups were performed using Kruskal–Wallis variance analysis followed by Dunn’s multiple comparison test. A receiver operating characteristic (ROC) curve was constructed to assess the performance of the plasma immune mediators in classifying the leprosy patients, the leprosy reaction patients, the HHC, and the NI healthy controls. The global accuracy was estimated based on the area under the ROC curve (AUC) along with sensitivity (Se) and specificity (Sp), which were employed as performance indices. In all cases, significance was considered at *p* < 0.05. Analysis of the fold change magnitude in the plasma immune mediator concentrations was performed for each clinical group according to the median values observed for the NI healthy controls. Changes in the plasma immune mediators were further assessed using the bi-dimensional scattering plot distribution of the fold change magnitude order *vs.* statistical significance. These analyses were carried out using Prism 8.0.2 software (GraphPad Software, San Diego, CA, USA).

Exploratory color map constructs were assembled to illustrate the ability of the plasma immune mediators to classify leprosy patients, leprosy reaction patients, HHC, and NI healthy controls using a color key (10th, 50th, and 90th of date distribution) to label distinct median values expressed in picograms per milliliter. These analyses were carried out using Microsoft Excel Office, version 2016.

DT algorithms were constructed to define the accuracy of the plasma immune mediators in classifying leprosy patients, leprosy reaction patients, HHC, and NI healthy controls. The “leave-one-out cross-validation” (LOOCV) was calculated as an additional performance index to generalize the results of a given statistical analysis into an independent dataset. These analyses were carried out using the WEKA software, version 3.6.11 (University of Waikato, New Zealand).

Single and combined stepwise analyses of the plasma immune mediators were compared to define the most accurate approach to classifying leprosy patients, leprosy reaction patients, HHC, and NI healthy controls.

## Results

### Fold change magnitude of the plasma immune mediators in leprosy patients, leprosy reaction patients, and household contacts compared with non-infected healthy controls

The overall profile of the immune mediators was assessed in the plasma samples from leprosy patients [L(PB) and L(MB)], leprosy reaction patients [T1LR and T2LR], and HHC compared with healthy controls (NI). The results are shown in **Table 2**. The data demonstrated that most plasma immune mediators were increased in L(PB), L(MB), T1LR, T2LR, and HHC compared with NI (**Table 2**).

**Table 2:** Overall profile of plasma immune mediators in leprosy patients, leprosy reactions patients, household contacts and non-infected healthy controls.

Immune Mediators		Study Groups					
		NI (n=18)	HHC (n=7)	L(PB) (n=14)	L(MB) (n=13)	T1LR (n=12)	T2LR (n=11)
Chemokines	CCL11	24.5 (12-35)	23.4 (4-48)	30.4 (8-59)	12.9 (5-45)	43.5 (17-61)	40.3 (13-63)
	CXCL8	1.4 (0.8-2)	97.9 ^a^ (68-193)	33.1 ^a^ (2-218)	54.2 ^a^ (2-299)	6.4 ^a^ (4-39)	8.2 ^a^ (3-137)
	CCL3	0.9 (0.6-0.9)	4.7 ^a^ (3-29)	3.2 ^a^ (1.3-7)	13.5 ^a,c^ (2-39)	5.4 ^a^ (2-8)	7.2 ^a,c^ (6-8)
	CCL4	4.9 (4-7)	26.8 ^a^ (11-39)	11.0 ^a,b^ (6-23)	19.7 ^a,c^ (12-39)	15.9 ^a^ (8-21)	19.6 ^a,c^ (16-25)
	CCL2	10.4 (7-22)	15.9 (11-19)	13.1 (8-22)	15.0 (6-37)	11.7 (4-31)	6.5 (3-43)
	CCL5	372.3 (225-463)	555.1 ^a,d^ (441-680)	502.2 ^a,d^ (353-652)	340.6 (263-433)	393.4 (252-523)	507.9 ^a,d^ (358-570)
	CXCL10	85.4 (53-124)	103.9 (88-135)	240.5 ^a,b^ (115-418)	326.6 ^a,b,c^ (194-1129)	415.3 ^a,b,c^ (221-1077)	445.1 ^a,b,c^ (162-793)
Pro-inflammatory Cytokines	IL-1β	0.2 (0.1-0.2)	0.5 ^a^ (0.1-0.9)	0.3 ^a^ (0.1-0.3)	0.2 ^a^ (0-0.3)	0.2 ^a^ (0.1-0.4)	0.2 ^a^ (0.1-0.3)
	IL-6	0.2 (0-0.3)	0.3 (0.1-0.6)	0.4 ^a^ (0.3-0.6)	0.5 ^a^ (0-0.7)	0.4 ^a^ (0.1-0.7)	0.5 ^a^ (0.2-0.5)
	TNF-α	1.6 (0.2-1.8)	3.4 ^a^ (2-3)	1.9 ^a^ (0.3-4)	2.5 ^a^ (1.4-9)	2.2 ^a^ (0.8-2)	4.0 ^a,b,e^ (2-7)
	IL-12	0.1 (0-0.2)	0.1 (0-1)	0.1 (0-3)	0.1 (0-1.3)	0.1 (0-0.2)	0.1 (0-0.9)
	IFN-γ	0.5 (0.2-1.3)	3.5 ^a^ (0.9-5)	1.2 ^a^ (0.8-5)	2.1 ^a^ (0-4)	2.6 ^a^ (0.8-3)	4.5 ^a,b,e^ (2-9)
	IL-15	47.5 (17-59)	49.9 (39-93)	57.2 (44-84)	66.6 (15-84)	66.5 ^a^ (14-107)	71 ^a^ (56-97)
	IL-17	4.6 (1-6)	5.1 ^a^ (3-12)	4.3 ^a^ (0.9-13)	3.6 (0.5-7)	7.2 ^a^ (1.6-16)	9.3 ^a,d^ (4-10)
Regulatory Cytokines	IL-1Ra	61.5 (47-82)	162.6 ^a^ (139-289)	105.9 ^a^ (61-323)	72.2 ^a^ (48-238)	142.5 ^a^ (84-232)	178.2 ^a^ (102-443)
	IL-4	1.4 (0.7-2)	0.9 (0.5-2)	1.7 (0.5-2)	1.1 (0.1-2)	2.0 (0.9-2)	1.5 (0.8-2)
	IL-5	8.0 (4-13)	5.5 (4-15)	9.6 (5-13)	6.8 (2-19)	11.9 ^a^ (8-18)	10 ^a^ (8-12)
	IL-9	5.8 (2-7)	8.7 ^a^ (5-9)	7.8 ^a^ (6-13)	7.6 ^a^ (4-22)	4.1 ^b,c,f^ (0.6-8)	10.4 ^a^ (7-19)
	IL-10	3.9 (2-6)	3.3 (1-6)	5.4 (3-16)	3.4 (1.4-7)	6.9 (2-11)	7.8 (2-9)
	IL-13	0.5 (0.2-1.1)	0.4 (0-1.2)	0.9 ^a^ (0.4-2)	0.9 (0.1-1.5)	1.1 ^a,b^ (0.4-1.9)	0.7 (0.4-1.4)
Growth Factors	FGF-basic	4.1 (2-7)	5.5 (4-6)	5.1 (2-7)	6.1 (4-9)	6.3 (3-10)	4.9 (1-6)
	PDGF	29.8 (17-68)	175.4 ^a^ (64-320)	81.5 ^a^ (32-138)	53.3 ^a,b^ (27-118)	53.7 ^a^ (29-132)	71.1 ^a,b^ (24-132)
	VEGF	6.1 (2-11)	13.0 (12-14)	9.1 (8-15)	5.7 (4-13)	10.7 (2-19)	7.8 (6-13)
	G-CSF	17.6 (8-33)	49.4 ^a^ (18-65)	34.7 ^a^ (19-56)	58.5 ^a^ (16-86)	41.2 ^a^ (18-62)	56.3 ^a^ (29-87)
	GM-CSF	0.1 (0-0.3)	0.1 (0-0.4)	0.2 (0-0.4)	0.2 (0-0.5)	0.4 ^a^ (0-0.6)	0.3 ^a^ (0.1-0.5)
	IL-2	1.8 (0.7-2)	2.9 ^a^ (0.9-3)	2.1 (1-3)	2.2 ^a^ (0.8-4)	2.3 ^a^ (1.8-4)	2.2 ^a^ (1.7-3)
	IL-7	3.3 (0.8-5)	1.6 (0.4-4)	6.2 ^a,b,d,f^ (4-19)	1.9 (0.6-5)	4.4 (2-8)	2.8 (0.9-4)

*NI, Non-infected Healthy Controls; HHC, Household Contacts; L(PB), Paucibacillary Leprosy Patients; L(MB), Multibacillary Leprosy Patients; T1LR, Patients with Type 1 Leprosy Reaction; T2LR, Patients with Type 2 Leprosy Reaction. The values are median of Immune Mediators in Plasma in pg/ml, and in parentheses are the 25% and 75% percentiles of the values. Comparative analyses between groups were carried out by Mann-Whitney test. Significant differences at p<0.05 are underscored by letters “a”, “b”, “c” “d” and “f” as compared to NI, HHC, L(PB), L(MB) and T1RL, respectively.

**Figure 1:**
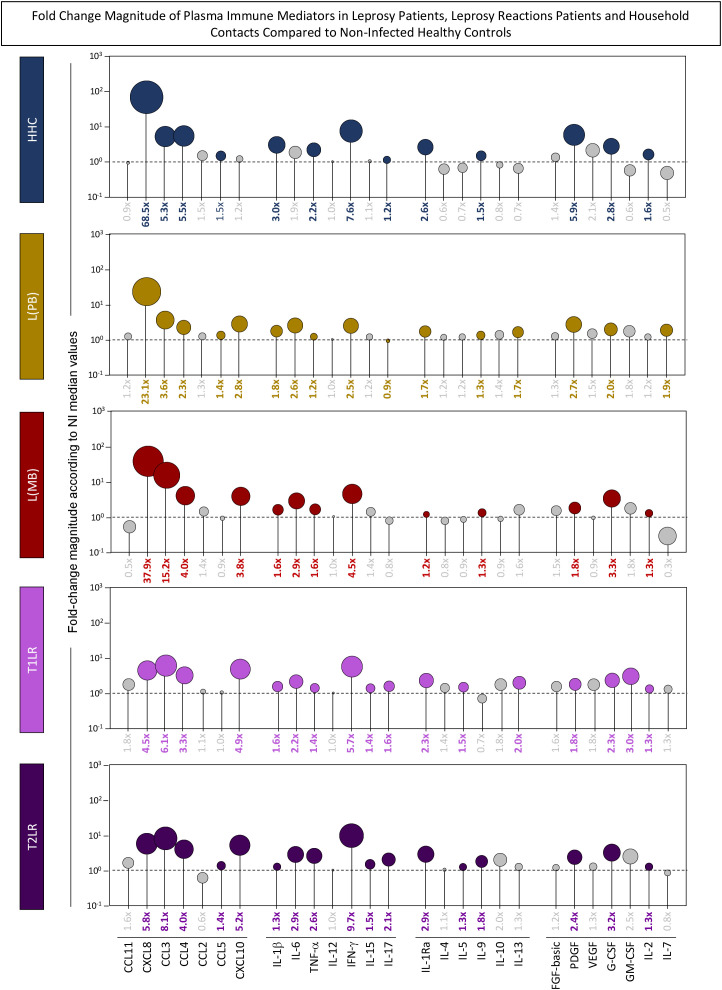
Fold change magnitude of the plasma immune mediators in leprosy patients, leprosy reaction patients, and household contacts compared with non-infected (NI) healthy controls. The levels of chemokines (CCL11, CXCL8, CCL3, CCL4, CCL2, CCL5, and CXCL10), pro-inflammatory cytokines (IL-1β, IL-6, TNF-α, IL-12, IFN-γ, IL-15, and IL-17), regulatory cytokines (IL-1Ra, IL-4, IL-5, IL-9, IL-10, and IL-13), and growth factors (FGF-basic, PDGF, VEGF, G-CSF, GM-CSF, IL-2, and IL-7) were measured in plasma samples collected from leprosy patients [L(PB) = 14, *brown rectangle*; L(MB) = 13, *dark red rectangle*], leprosy reaction patients (T1LR = 12, *violet rectangle*; T2LR = 11, *purple rectangle*), household contacts (HHC = 7, *dark blue rectangle*), and NI healthy controls (NI = 18). The levels of plasma immune mediators were quantified using high-throughput multiplex microbead array as described in the *Materials and methods*. The levels of the plasma immune mediators in each study group were compared with those in NI using the Mann–Whitney test. Significance was considered at *p* < 0.05. The results shown in lollipop charts are expressed as median fold changes according to NI. *Colored nodes* and fold change values represent the plasma immune mediators with significant differences compared with NI. *Gray nodes* represent the plasma immune mediators with non-significant differences compared with NI. Node sizes are proportional to the fold change values.

To further characterize the profiles of the plasma immune mediators among groups, the magnitude of fold change was calculated for each analyte according to the median values observed for the NI healthy controls. The results are presented in **Figure 1**. Data analysis of the fold change values was carried out using color nodes to highlight the plasma immune mediators with significant differences compared with NI. Gray nodes represent the plasma immune mediators with no significant differences compared with NI. The results demonstrated that HHC presented increased fold changes for 13 out of 27 (48%) immune mediators, ranging from 68.5× to 1.2×, with CXCL8 > IFN-γ > PDGF > CCL4 > CCL3 > IL-1β > G-CSF > IL-1Ra > TNF-α > IL-2 > IL-9 > CCL5 > IL-17. Analysis of the L(PB) group showed increased fold change magnitudes for 15 out of 27 molecules (56%), ranging from 23.1× to 1.2×, with CXCL8 > CCL3 > CXCL10 > PDGF > IL-6 > IFN-γ > CCL4 > G-CSF > IL-7 > IL-1β > IL-1Ra > IL-13 > CCL5 > IL-9 > TNF-α. The L(MB) group presented increased levels of 13 out of 27 immune mediators (48%), which ranged from 37.9× to 1.2×, with CXCL8 > CCL3 > IFN-γ > CCL4 > CXCL10 > G-CSF > IL-6 > PDGF > TNF-α > IL-1β > IL-9 > IL-2 > IL-1Ra. In general, the leprosy reaction patients exhibited an overall increase in plasma immune mediators with lower magnitude order. The T1LR group presented increased levels for 17 out of 27 immune mediators (63%), ranging from 6.1× to 1.3×, with CCL3 > IFN-γ > CXCL10 > CXCL8 > CCL4 > GM-CSF > G-CSF > IL-1Ra > IL-6 > IL-13 > PDGF > IL-17 > IL-1β > IL-5 > TNF-α > IL-15 > IL-2. The T2LR group showed increased fold change magnitudes of 17 out of 27 immune mediators (63%), ranging from 9.7× to 1.5×, with IFN-γ > CCL3 > CXCL8 > CXCL10 > CCL4 > G-CSF > IL-1Ra > IL-6 > TNF-α > PDGF > IL-17 > IL-9 > IL-15 > CCL5 > IL-2 > IL-1β > IL-5 (**Figure 1**).

### Bi-dimensional scattering profiles of the plasma immune mediators in leprosy patients and non-infected healthy controls

**Figure 2:**
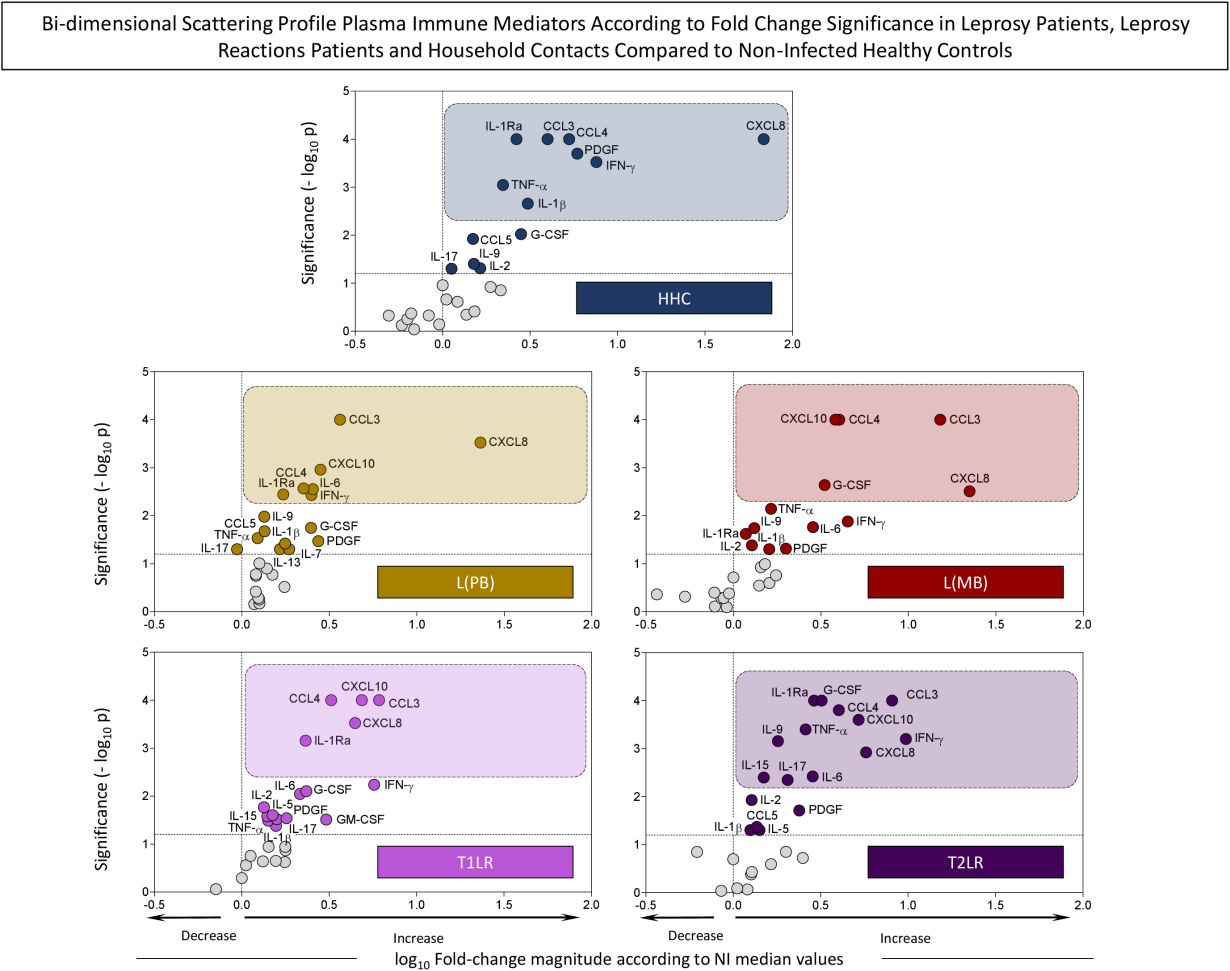
Bi-dimensional scattering profiles of the plasma immune mediators according to their fold change magnitude and significance in leprosy patients, leprosy reaction patients, and household contacts compared with non-infected (NI) healthy controls. The levels of chemokines (CCL11, CXCL8, CCL3, CCL4, CCL2, CCL5, and CXCL10), pro-inflammatory cytokines (IL-1β, IL-6, TNF-α, IL-12, IFN-γ, IL-15, and IL-17), regulatory cytokines (IL-1Ra, IL-4, IL-5, IL-9, IL-10, and IL-13), and growth factors (FGF-basic, PDGF, VEGF, G-CSF, GM-CSF, IL-2, and IL-7) were measured in plasma samples collected from leprosy patients [L(PB) = 14, *brown rectangle*; L(MB) = 13, *dark red rectangle*], leprosy reaction patients (T1LR = 12, *violet rectangle*; T2LR = 11, *purple rectangle*), household contacts (HHC = 7, *dark blue rectangle*), and NI healthy controls (NI = 18). The levels of plasma immune mediators were quantified using high-throughput multiplex microbead array as described in *Materials and methods*. The levels of the plasma immune mediators in each study group were compared with those in NI using the Mann–Whitney test. Significance was considered at *p* < 0.05. The results shown are expressed as bi-dimensional scattering distribution of the log_10_ fold change magnitude according to NI by −log_10_ of *p*-values. The *dashed line across the y-axis* indicates the significance threshold (−log_10_ 0.05 = 1.301). The *dashed line across the x-axis* defines the decrease or increase of the log_10_ fold changes. *Colored dots* represent plasma immune mediators with significant differences compared with NI. Plasma immune mediators exhibiting significance with *p* < 0.01 (−log_10_ = 2) are underscored by *colored rectangles*.

To build the bi-dimensional scattering profile graphs, the fold change magnitudes in the plasma immune mediator concentrations were calculated for each studied clinical group considering the median values observed for the NI healthy group. Subsequently, the changes in the plasma immune mediators were further assessed using bi-dimensional scattering plot distribution of the fold change magnitude order *vs.* statistical significance. The results are presented in **Figure 2**. Plasma immune mediators with significant differences at *p* < 0.05 (−log10 0.05 = 1.301) compared with NI were underscored using colored dots. Further analysis of the fold change significance considering the threshold of *p* < 0.01 (−log10 = 2) allowed the selection of plasma immune mediators with putative probability of identifying the intrinsic profile of each clinical group (**Figure 2**, colored rectangles).

Based on this criterion, eight plasma immune mediators (8/27, 30%) were selected as attributes with higher significance (*p* < 0.01) in HHC: CXCL8, IFN-γ, PDGF, CCL4, CCL3, IL-1β, IL-1Ra, and TNF-α. A set of seven immune mediators (7/27, 30%) had higher significance (*p* < 0.01) in L(PB), namely, CXCL8, CCL3, CXCL10, IL-6, IFN-γ, CCL4, and IL-1Ra. For L(MB), CXCL8, CCL3, CCL4, CXCL10, and G-CSF (5/27, 19%) presented higher significance values (*p* < 0.01). The analysis of the leprosy reaction patients demonstrated that while CCL3, CXCL10, CXCL8, CCL4, and IL-1Ra (5/27, 19%) presented higher significance (*p* < 0.01) for T1LR, a large set of attributes (12/27, 44%) were selected for T2LR, which included IFN-γ, CCL3, CXCL8, CXCL10, CCL4, G-CSF, IL-1Ra, IL-6, TNF-α, IL-17, IL-9, and IL-15 (**Figure 2**).

### Performance of the plasma immune mediators in classifying leprosy patients, leprosy reaction patients, household contacts, and non-infected healthy controls

To define the applicability of the plasma immune mediators in classifying HHC and the distinct clinical forms of leprosy, ROC curve analysis was performed. The performance indices (AUC, Se, and Sp) are presented in **Table 3**. Data analysis demonstrated that CXCL8, CCL3, CCL4, TNF-α, IL-1Ra, PDGF, IFN-γ, G-CSF, IL-1β, IL-2, CCL5, and IL-9 are attributes with moderate/high performance (AUC > 0.70) in classifying NI *vs.* HHC. CCL3 was the single attribute with moderate/high performance in classifying L(PB) *vs.* L(MB). Five plasma immune mediators (IL-9, TNF-α, IFN-γ, FGF-basic, and CCL4) presented moderate/high performance in classifying T1LR *vs.* T2LR (**Table 3**).

**Table 3:** Performance of plasma immune mediators to classify leprosy patients, leprosy reactions patients, household contacts and non-infected healthy controls.

Parameter (Cut-Off)	NI vs HHC			Parameter (Cut-Off)	L(PB) vs L(MB)			Parameter (Cut-Off)	T1LR vs T2LR		
	AUC (95%CI)	Se (95%CI)	Sp (95%CI)		AUC (95%CI)	Se (95%CI)	Sp (95%CI)		AUC (95%CI)	Se (95%CI)	Sp (95%CI)
**CXCL8** **(>5.9)**	**0.98** **(0.9-1.0)**	**100** **(59-100)**	**88.9** **(65-98)**	**CCL3** **(>12.0)**	**0.70** **(0.5-0.9)**	**53.9** **(29-81)**	**100** **(78-100)**	**IL-9** **(>9.0)**	**0.87** **(0.7-1.0)**	**72.7** **(43-90)**	**91.7** **(65-99)**
**CCL3** **(>1.0)**	**0.95** **(0.9-1.0)**	**100** **(59-100)**	**88.9** **(65-98)**	CCL4 (>7.5)	0.69 (0.5-0.9)	92.3 (64-99)	42.9 (18-71)	**TNF-α** **(>3.1)**	**0.77** **(0.6-1.0)**	**63.6** **(31-89)**	**83.3** **(52-97)**
**CCL4** **(>6.8)**	**0.95** **(0.9-1.0)**	**100** **(59-100)**	**77.8** **(52-94)**	CCL5 (≤449.1)	0.69 (0.5-0.9)	76.9 (46-95)	64.3 (35-87)	**IFN-γ** **(>3.4)**	**0.73** **(0.5-0.9)**	**54.5** **(24-83)**	**91.7** **(62-99)**
**TNF-α** **(>2.4)**	**0.90** **(0.8-1.0)**	**85.7** **(42-98)**	**94.4** **(73-99)**	CXCL10 (>160.7)	0.65 (0.4-0.9)	92.3 (64-99)	42.9 (18-71)	**FGF-basic** **(≤1.2)**	**0.71** **(0.5-0.9)**	**72.7** **(39-94)**	**66.7** **(35-90)**
**IL-1Ra** **(>107.6)**	**0.87** **(0.7-1.0)**	**85.7** **(42-98)**	**94.4** **(73-99)**	IL-10 (≤3.44)	0.62 (0.4-0.8)	53.8 (25-81)	71.4 (42-91)	**CCL4** **(>15.9)**	**0.70** **(0.5-0.9)**	**90.9** **(59-99)**	**58.3** **(28-85)**
**PDGF** **(>107.6)**	**0.87** **(0.7-1.0)**	**71.4** **(29-96)**	**100** **(81-100)**	TNF-α (>1.6)	0.60 (0.4-0.8)	76.9 (46-95)	50 (23-77)	G-CSF (>22.8)	0.67 (0.4-0.9)	100 (71-100)	33.3 (10-65)
**IFN-γ** **(>2.9)**	**0.82** **(0.6-1.0)**	**71.4** **(29-96)**	**100** **(81-100)**	IL-17 (≤9.9)	0.59 (0.4-0.8)	92.3 (64-99)	35.7 (13-65)	CCL3 (>5.8)	0.66 (0.4-0.9)	90.9 (59-99)	58.3 (28-85)
**G-CSF** **(>44.1)**	**0.79** **(0.6-1.0)**	**57.1** **(19-90)**	**94.4** **(73-99)**	G-CSF (>52.0)	0.59 (0.4-0.8)	61.5 (32-86)	71.4 (42-91)	IL-4 (≤2.1)	0.64 (0.4-0.9)	81.8 (48-97)	50 (21-79)
**IL-1β** **(>0.3)**	**0.78** **(0.5-1.0)**	**71.4** **(29-96)**	**94.4** **(73-99)**	IL-1Ra (≤290.4)	0.58 (0.4-0.8)	92.3 (64-99)	35.7 (13-65)	CCL5 (>424.9)	0.63 (0.4-0.9)	77.8 (40-97)	58.3 (28-85)
**IL-2** **(>2.8)**	**0.76** **(0.5-1.0)**	**57.1** **(19-90)**	**94.4** **(73-99)**	IL-4 (≤1.3)	0.58 (0.4-0.8)	69.2 (39-91)	57.1 (29-82)	IL-1Ra (>237)	0.61 (0.4-0.8)	45.5 (17-77)	83.3 (52-97)
**CCL5** **(>552.4)**	**0.74** **(0.5-1.0)**	**71.4** **(29-96)**	**88.9** **(65-98)**	PDGF (≤67.7)	0.57 (0.3-0.8)	76.9 (46-95)	50 (23-77)	VEGF (>3.2)	0.60 (0.3-0.9)	90 (56-98)	58.3 (28-85)
**IL-9** **(>8.2)**	**0.71** **(0.5-0.9)**	**57.1** **(19-90)**	**83.3** **(59-96)**	IL-1β (≤0.1)	0.56 (0.3-0.8)	38.5 (14-68)	78.6 (49-95)	IL-1β (≤0.2)	0.59 (0.3-0.8)	72.7 (39-94)	66.7 (35-90)
IL-12 (≤0.01)	0.69 (0.5-0.9)	100 (59-100)	38.9 (17-64)	CCL11 (≤15.7)	0.56 (0.3-0.8)	61.5 (32-86)	64.3 (35-87)	IL-13 (≤1.7)	0.59 (0.4-0.8)	100 (71-100)	25 (6-57)
IL-6 (>0.3)	0.67 (0.4-0.9)	57.1 (19-90)	77.8 (52-94)	IL-13 (≤0.2)	0.56 (0.3-0.8)	38.5 (14-68)	78.6 (49-95)	IL-17 (>4.2)	0.58 (0.3-0.8)	90.9 (59-99)	41.7 (15-72)
CXCL10 (>69.5)	0.66 (0.4-0.9)	100 (59-100)	44.4 (22-69)	CXCL8 (>253.5)	0.55 (0.3-0.8)	30.8 (9-61)	92.9 (66-99)	IL-5 (≤16.3)	0.56 (0.3-0.8)	90.9 (59-99)	33.3 (10-65)
IL-17 (>7.3)	0.64 (0.4-0.9)	42.9 (10-81)	88.9 (65-98)	CCL2 (>30.7)	0.55 (0.3-0.8)	30.8 (9-61)	92.9 (66-99)	IL-6 (>0.2)	0.56 (0.3-0.8)	90.9 (59-99)	33.3 (10-65)
IL-7 (>0.01)	0.64 (0.4-0.9)	85.7 (42-98)	52.9 (28-77)	IL-12 (≤1.8)	0.55 (0.3-0.8)	92.3 (64-99)	28.6 (9-58)	GM-CSF (≤0.8)	0.54 (0.3-0.8)	100 (71-100)	16.7 (3-48)
IL-15 (>84.8)	0.63 (0.4-0.9)	28.6 (5-71)	100 (81-100)	FGF-basic (≤0.01)	0.54 (0.3-0.8)	53.8 (25-81)	64.3 (35-87)	IL-10 (≤11.0)	0.53 (0.3-0.8)	90.9 (59-99)	25 (6-57)
CCL2 (>11.5)	0.62 (0.4-0.9)	85.7 (42-98)	61.1 (36-83)	IL-15 (>63.4)	0.53 (0.3-0.8)	53.8 (25-81)	64.3 (35-87)	IL-2 (>0.9)	0.53 (0.3-0.8)	100 (71-100)	16.7 (3-48)
IL-13 (≤0.4)	0.61 (0.3-0.9)	71.4 (29-96)	66.7 (41-87)	IL-5 (≤6. 8)	0.52 (0.3-0.8)	53.8 (25-81)	71.4 (42-91)	CXCL8 (>120.5)	0.53 (0.3-0.8)	27.3 (6-61)	91.7 (62-99)
VEGF (≤0.01)	0.60 (0.3-0.9)	71.4 (29-96)	61.1 (36-83)	IFN-γ (≤0.7)	0.52 (0.3-0.8)	38.5 (14-68)	92.9 (66-99)	CCL2 (≤6.5)	0.53 (0.3-0.8)	54.5 (24-83)	75 (43-94)
IL-10 (≤1.0)	0.58 (0.3-0.8)	28.6 (5-71)	94.4 (73-99)	IL-6 (≤0.03)	0.51 (0.3-0.7)	30.8 (9-61)	92.9 (66-99)	CCL11 (≤44.0)	0.52 (0.3-0.8)	63.6 (31-89)	50 (21-79)
IL-4 (≤0.9)	0.55 (0.3-0.8)	57.1 (19-90)	72.2 (47-90)	IL-2 (>3.5)	0.51 (0.3-0.7)	30.8 (9-61)	85.7 (57-98)	IL-15 (>0.01)	0.51 (0.3-0.8)	100 (71-100)	25 (6-57)
GM-CSF (>0.28)	0.54 (0.3-0.8)	42.9 (10-81)	72.2 (47-90)	IL-7 (≤4.2)	0.51 (0.3-0.8)	83.3 (52-97)	38.5 (14-68)	PDGF (>36.5)	0.51 (0.3-0.8)	63.6 (31-89)	50 (21-79)
FGF-basic (>4.2)	0.54 (0.3-0.8)	28.6 (5-71)	83.3 (59-96)	GM-CSF (>0.8)	0.51 (0.3-0.7)	15.4 (2-46)	100 (77-100)	IL-7 (>0.05)	0.51 (0.3-0.8)	81.8 (48-97)	41.7 (15-72)
IL-5 (≤5.5)	0.52 (0.2-0.8)	57.1 (19-90)	72.2 (47-90)	IL-9 (>15.5)	0.51 (0.3-0.8)	27.3 (6-61)	92.9 (66-99)	CXCL10 (>348.9)	0.51 (0.3-0.8)	63.6 (31-89)	50 (21-79)
CCL11 (≤12.6)	0.50 (0.2-0.8)	42.9 (10-81)	77.8 (52-94)	VEGF (>2.1)	0.50 (0.3-0.7)	83.3 (52-97)	42.9 (18-71)	IL-12 (>0.3)	0.50 (0.3-0.7)	27.3 (6-61)	91.7 (62-99)

*NI, Non-infected Healthy Controls; HHC, HouseHold Contacts; L(PB), Paucibacillary Leprosy Patients; L(MB), Multibacillary Leprosy Patients; T1LR, Patients with Type 1 Leprosy Reaction; T2LR, Patients with Type 2 Leprosy Reaction. Performance of soluble mediator to classify group of subjects was assessed by Receiver Operating Characteristics (ROC) curves. Global accuracy was estimated by Area Under the Curve (AUC). Sensitivity (Se) and Specificity (Sp) were determined at specific Cut-Off (expressed in pg/mL) indicated by the ROC curves. CI, confidence interval. Soluble mediators with moderate/high AUC (>0.70) are highlighted by bold underline format.

**Figure 3:**
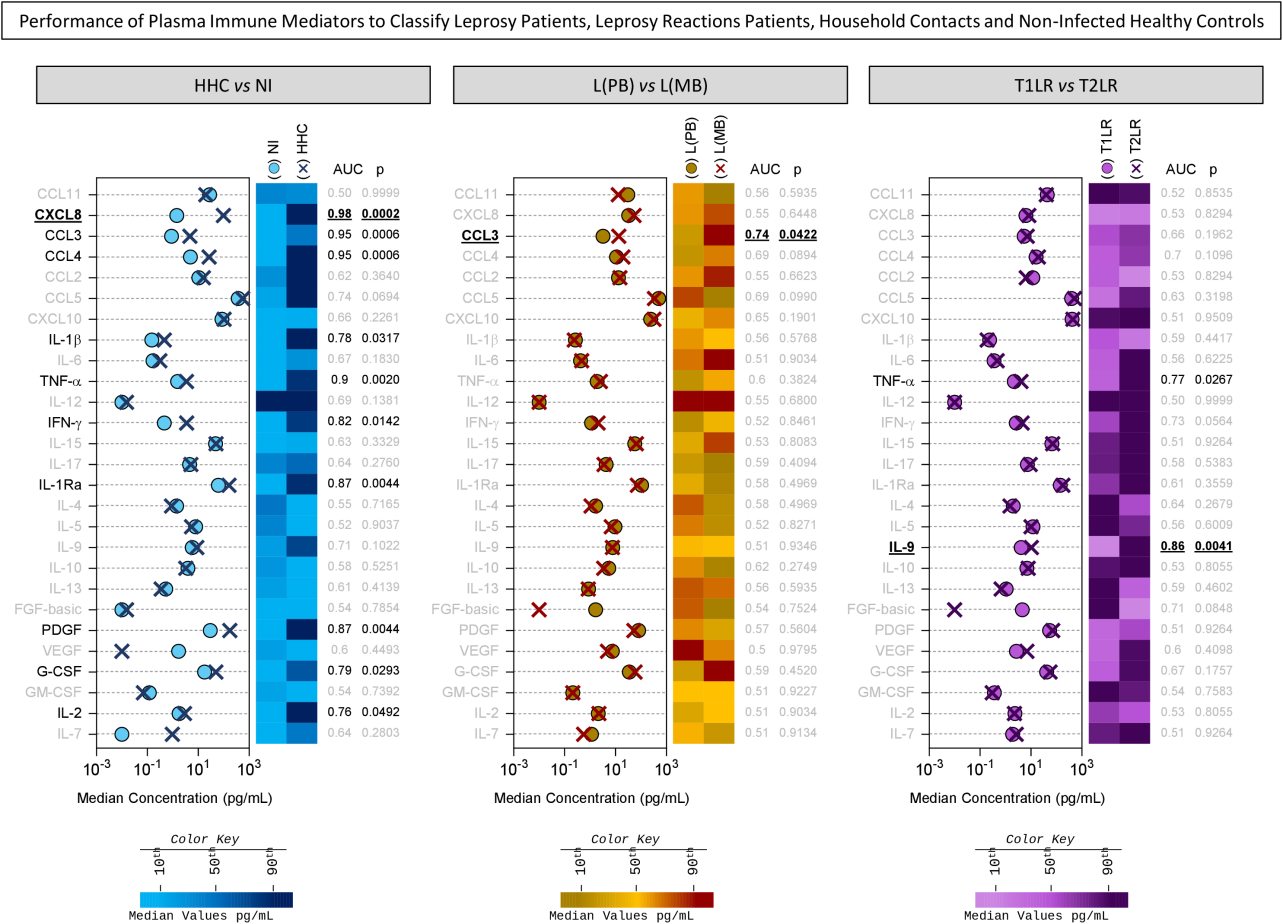
Performance of plasma immune mediators in classifying leprosy patients, leprosy reaction patients, household contacts (HHC), and non-infected (NI) healthy controls. The performance of chemokines (CCL11, CXCL8, CCL3, CCL4, CCL2, CCL5, and CXCL10), pro-inflammatory cytokines (IL-1β, IL-6, TNF-α, IL-12, IFN-γ, IL-15, and IL-17), regulatory cytokines (IL-1Ra, IL-4, IL-5, IL-9, IL-10, and IL-13), and growth factors (FGF-basic, PDGF, VEGF, G-CSF, GM-CSF, IL-2, and IL-7) was assessed using a receiver operating characteristic (ROC) curve to classify HHC (*dark blue cross*) from NI healthy controls (NI = 18, *light blue circle*), the paucibacillary [L(PB) = 14, *brown circle*] from the multibacillary leprosy patients [– L(MB) = 13, *dark red cross*], and the type 1 (T1LR = 12, *violet circle*) from the type 2 leprosy reaction patients (T2LR = 11, *purple cross*). The levels of the plasma immune mediators were quantified using high-throughput multiplex microbead array as described in *Materials and methods*. The data are presented as median concentration (in picograms per milliliter). The area under the ROC curve (AUC) was used as an indicator of global accuracy to classify the clinical groups. Significance was considered at *p* < 0.05. The plasma immune mediators with significant global accuracy are underscored in *black*, and those with higher AUC are highlighted with a *bold underline*. Color maps were constructed based on the median concentration value of each plasma immune mediator to further illustrate their ability to classify the clinical subgroups using the color keys as provided in the figure.

Additional analysis was carried out to identify among the preselected attributes the plasma immune mediators with significant performance in classifying HHC and the distinct clinical forms of leprosy. The results are presented in **Figure 3**. The results of the data analysis, reported as the median plasma concentration (in picograms per milliliter), were used to demonstrate the ability of the plasma immune mediators to classify the clinical groups [NI *vs.* HHC, L(PB) *vs.* L(MB), and T1LR *vs.* T2LR]. The AUC values were used as indicators of global accuracy, with significance considered at *p* < 0.05. The analysis confirmed that, except for CCL5, all preselected attributes displayed significant ability to segregate NI from HHC (CXCL8, CCL3, CCL4, TNF-α, IL-1Ra, PDGF, IFN-γ, G-CSF, IL-1β, IL-2, and IL-9), with CXCL8 presenting the most outstanding performance (AUC = 0.98, *p* = 0.0002). The data confirmed the significance of CCL3 (AUC = 0.74, *p* = 0.0422) in classifying L(PB) from L(MB). Among the preselected attributes, although TNF-α displayed significant performance, IL-9 showed a higher ability to classify T1LR from T2LR (AUC = 0.86, *p* = 0.0041) (**Figure 3**).

### Decision tree algorithms for the use of plasma immune mediators to classify leprosy patients, leprosy reaction patients, household contacts, and healthy controls

**Figure 4:**
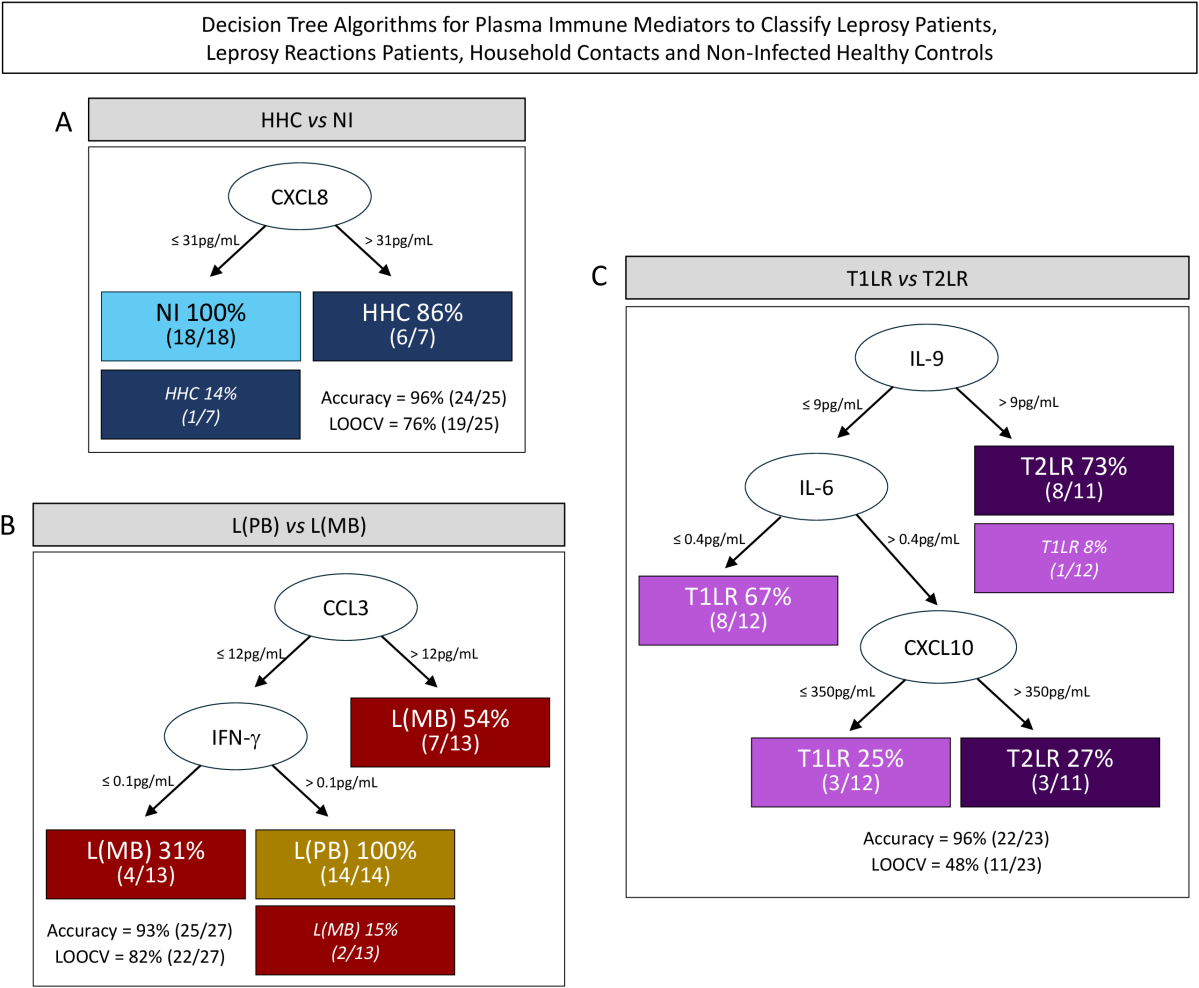
Decision tree algorithms for the use of plasma immune mediators to classify leprosy patients, leprosy reaction patients, household contacts (HHC), and healthy controls. A decision tree was constructed using the plasma immune mediators to create algorithms for the classification of HHC (HHC = 7, *dark blue rectangle*) from non-infected (NI) healthy controls; (NI = 18, *light blue rectangle*) **(A)**; paucibacillary [L(PB) = 14, *brown rectangle*] from multibacillary leprosy patients [L(MB) = 13, *dark red rectangle*] **(B)**; and type 1 (T1LR = 12, *purple rectangle*) from type 2 leprosy reaction patients (T2LR = 11, *indigo rectangle*) **(C)**. The *roots* (CXCL8, CCL3, and IL-9) and the *branch nodes* (IFN-γ, IL-6, and CXCL10) were used to create leaves for each subgroup classification, displaying the number of subjects ranked on each pathway considering the cutoff edges defined by machine learning models as provided for each branch. The number of misclassifications is indicated in *parentheses*. The accuracy and the “leave-one-out-cross-validation” (LOOCV) values, considered as performance indices, are also provided.

Systems biology approaches were employed to create DT algorithms and use them to classify HHC and the distinct clinical forms of leprosy based on differential concentrations of plasma immune mediators. The results are presented in **Figure 4**. The data demonstrated that CXCL8 is a single root attribute capable of differentiating NI *vs.* HHC using a plasma concentration of 31 pg/ml as a cutoff, which yielded high accuracy (96%) and moderate cross-validation (LOOCV = 76%). This DT was able to correctly classify 100% (18/18) of the individuals from the NI group and 86% (6/7) of the HHC, with only one misclassification (1/25, 4%) (**Figure 4A**).

The DT algorithm proposed to classify the patients with leprosy into subgroups according to the operational classification showed CCL3 as a root attribute (cutoff = 12 pg/ml) and IFN-γ (cutoff = 0.1 pg/ml) as a branch biomarker for classifying L(MB) and L(PB), which had high accuracy (93%) and cross-validation (LOOCV = 82%). This DT was able to correctly classify 100% (14/14) of the patients from the L(PB) group and 85% (11/13) of those from the L(MB) group, with two misclassifications (2/13, 15%) (**Figure 4B**).

The algorithm assembled to classify patients with leprosy into subgroups according to the presence of leprosy reactions proposed the use of IL-9 as the root attribute (cutoff = 9 pg/ml) followed by IL-6 (cutoff = 0.4 pg/ml) and CXCL10 (cutoff = 350 pg/ml) as branch biomarkers for classifying T1RL and T2RL. This algorithm correctly classified 100% (11/11) of the patients from the T2RL group and 92% (11/12) of the patients from the T1LR group, with only one misclassification (1/23, 4%) (**Figure 4C**).

### Single and combined stepwise analyses of the plasma immune mediators to classify leprosy patients, leprosy reaction patients, household contacts, and non-infected healthy controls

**Figure 5:**
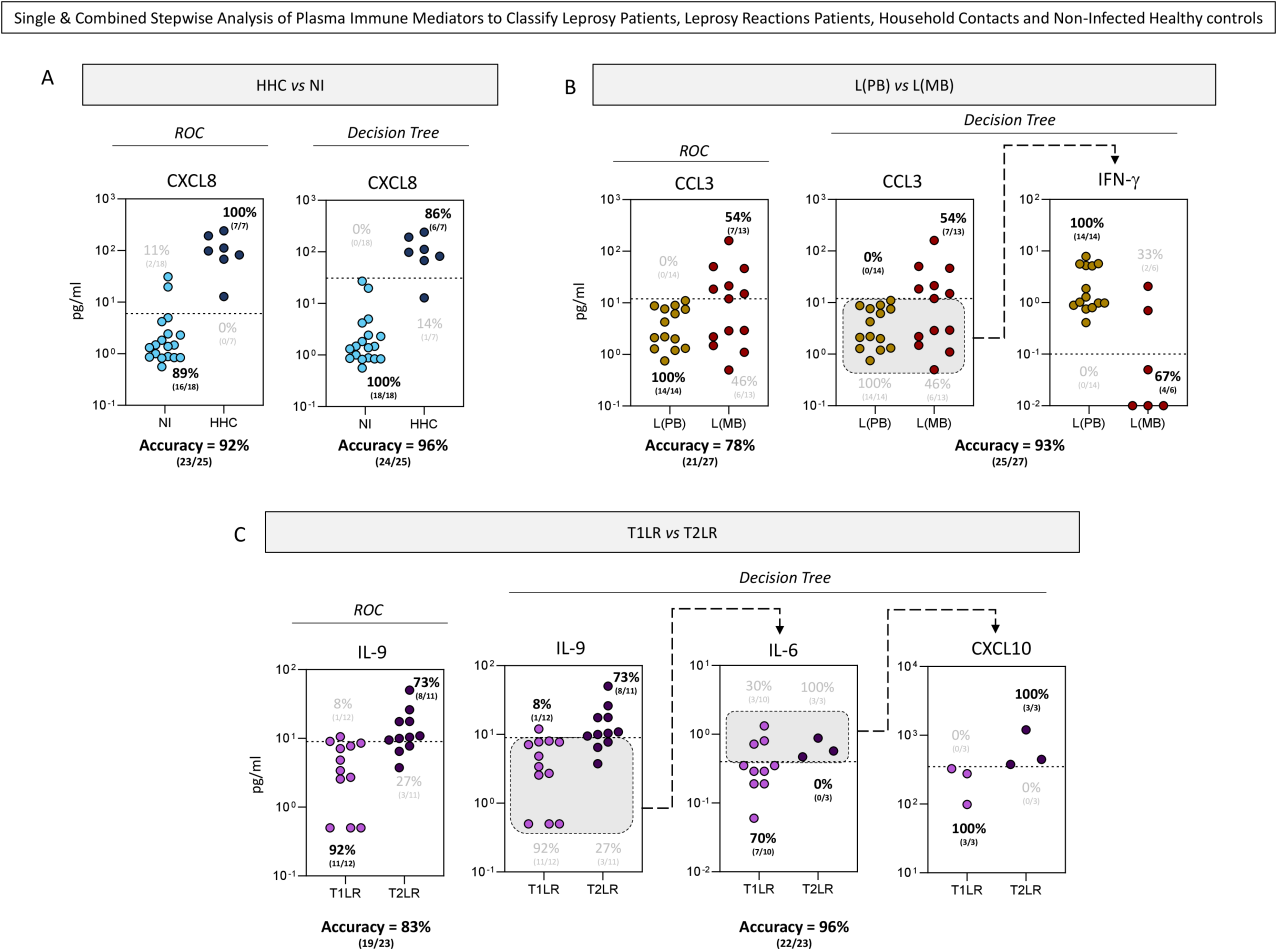
Single and combined stepwise analyses of the plasma immune mediators for the classification of leprosy patients, leprosy reaction patients, household contacts (HHC), and non-infected (NI) healthy controls. Single and combined stepwise analyses of the plasma immune mediators assessed the classification of HHC (HHC = 7, *dark blue circle*) from NI healthy controls (NI = 18, *light blue circle*) **(A)**; paucibacillary [L(PB) = 14, *brown circle*] from multibacillary leprosy patients [L(MB) = 13, *dark red circle*] **(B)**; and type 1 (T1LR = 12, *purple circle*) from type 2 leprosy reaction patients (T2LR = 11, *indigo circle*) **(C)**. Single-step analysis of the plasma immune mediators (CXCL8, CCL3, and IL-9) was carried out using preselected attributes based on the area under the receiver operating characteristic (ROC) curve (AUC). Combined stepwise analysis of the plasma immune mediators was carried out with additional attributes (IFN-γ, IL-6, and CXCL10) indicated by the decision tree algorithms using machine learning models. The scatter plot displays the discriminant analysis of the clinical subgroups using single and combined stepwise approaches. *Dotted lines* represent the cutoffs previously selected by the ROC curve or decision tree analysis. The frequencies of the samples segregated by the cutoffs are provided on each scatter plot. *Gray backgrounds* underscore the samples selected for the sequential stepwise analysis. The proportion of correct results (accuracy) from the single and combined stepwise approaches is also provided.

The accuracy of the single-step classification using the preselected attributes defined by the ROC curve analysis was compared with that of the combined stepwise analysis proposed by the DT algorithms. The results are presented in **Figure 5**.

Overall, the use of DT algorithms improved the performance of the analysis of the plasma immune mediators classifying HHC and the distinct clinical forms of leprosy (**Figure 5**). The performance of the single-step analysis in classifying NI and HHC demonstrated that the cutoffs proposed by the DT algorithm yielded higher accuracy (96%) compared with the ROC curve (92%) (**Figure 5A**). Comparative analysis of the single (CCL3) and combined stepwise (CCL3 followed by IFN-γ) analyses in classifying L(PB) and L(MB) also demonstrated the superior accuracy of the DT algorithm (93%) compared with the ROC curve (78%) (**Figure 5B**). The combined stepwise analysis (IL-9 followed by IL-6 and CXCL10) showed an enhanced accuracy in classifying T1LR and T2LR (87%) compared with the single analysis proposed by the ROC curve (83%) (**Figure 5C**).

## Discussion

Understanding the immunological events associated with the clinical manifestations involved in leprosy is important to support the establishment of new strategies for the clinical management of patients with leprosy and to monitor the progression of the disease and the development of leprosy reactions (26). As leprosy is a spectral disease, in many situations presenting an extremely challenging clinical diagnosis, there is a need for the identification of biomarkers that have clinical value in screening patients with leprosy. During the chronic course of leprosy, acute inflammatory manifestations could occur and clinically evolve into neural and cutaneous lesions, called leprosy reactions. These episodes affect a large proportion of patients with leprosy and may occur before diagnosis, as well as even after the end of treatment. Episodes of leprosy reactions are triggered by acute inflammatory events in response to the presence of *M. leprae* antigens, which leads to changes in the plasma levels of several immune response mediators; therefore, these mediators could be used as diagnostic and prognostic markers of these events.

Recent studies have sought to identify immunological biomarkers that could be used in the development of rapid tests to be utilized as tools in the diagnosis/prognosis of diseases. As an example, the interferon gamma release assay (IGRA) is already used for the diagnosis of patients with tuberculosis. In addition, IGRA has been suggested for the complementary diagnosis of paucibacillary patients (27). Another example of rapid testing is the up-converting phosphor lateral flow assay (UCP-LFA), which is capable of detecting the cytokine IL-10, the chemokines CXCL10 and CCL4, and anti-phenolic glycolipid I (PGL-I) IgM antibodies. These tests have already been validated in cohorts in Brazil, China, and Ethiopia and have shown a high correlation with the correlate ELISA method (23, 25). In the case of leprosy, we believe that the mediators CXCL8, CCL3, CXCL10, IL-9, IL-6, and IFN-γ could be considered as potential biomarkers for the future development of rapid assays that could assist in the diagnosis and prognosis of leprosy and leprosy reactions.

Another important data generated by our work was the description of the levels of 27 immune response mediators present in the plasma from the Household contact (HHC). The results showed that the HHC group had a predominantly pro-inflammatory profile with increased levels of chemokines (CXCL8, CCL3, CCL4, and CXCL10), pro-inflammatory cytokines (IL-1β, TNF-α, and IFN-γ), modulatory cytokines (IL1Ra and IL-9), and growth factors (PDGF, G-CSF, and IL-2) when compared with the levels of circulating biomarkers present in the NI healthy controls. These results corroborate other studies that showed alterations in the different plasma immune mediators in HHC and suggest that they may present a subclinical disease (28–30). Reinforcing this hypothesis, Araújo et al. (28) detected genetic material of the bacillus in 4.7% of nasal swabs and the presence of anti-PGL-I antibodies in 13.3% of HHC. The authors also described that HHC may be actively involved in the transmission chain of leprosy.

In this work, a high-throughput Luminex Bio-Plex microbead immunoassay was used for the quantitative measurements of the 27 immunological mediators in the plasma of patients with different clinical forms of leprosy. After quantifying the mediators, statistical tools and algorithmic methods were employed to identify the biomarkers that may be associated with leprosy and/or leprosy reactions and could help clinical doctors in the diagnosis and prognosis of patients with leprosy. These algorithmic methods have already been used in various studies of different diseases such as coronavirus disease 2019 (COVID-19), yellow fever, and human T-lymphotropic virus (HTLV), among others (31–33). Although this method has not yet been applied in the clinic, it is capable of identifying potential immunological markers that could be used for diagnosis and prognosis in these diseases. Overall, the results point out that most of the plasma immune mediators were increased in all clinical groups compared with NI.

Analyses based on the magnitude order of the increase identified a higher number of immune mediators which were increased, six of these being the best biomarkers—the chemokines CXCL10, CCL3, and CXCL8; the pro-inflammatory cytokines IFN-γ and IL-6; and the regulatory cytokine IL-9—which showed greater clinical relevance and possible applicability in the diagnosis of leprosy. Thus, using the classification tree, it was possible to categorize patients according to the combination of the plasma concentrations of two or more of these selected biomarkers.

The first group of plasma immune response mediators evaluated in this work comprised chemokines. These mediators are protein molecules that can recruit and activate specific subpopulations of leukocytes into sites of tissue injury and can act as potent mediators or regulators of inflammation. Using immunohistochemistry, Kirkaldy et al. (15) determined the expression of the chemokines CCL2, CCL5, and CXCL8 in lesions of different clinical forms of leprosy. According to the authors, the chemokine CXCL8, which is essential in recruiting monocytes and lymphocytes into the site of infection, favors the regression of lesions in patients with leprosy. Hassan et al. (17) found a reduction of CCL2 and an increase of CCL5 in the serum of the patients in the leprosy group when compared with the healthy control group. The increased CCL5 levels were observed mainly in paucibacillary patients than in multibacillary patients. Other authors also observed that paucibacillary and multibacillary patients had increased concentrations of the chemokines CCL2, CCL3, CCL11, CXCL8, CXCL9, and CXCL10 in plasma compared with the healthy group (18, 34, 35). According to Mendonça et al. (21), the plasma levels of the chemokines CCL2, CCL3, and CCL11 can be detected in patients with leprosy lesions, which remained unchanged after leprosy treatment. These results partially corroborate the findings of our study, which showed an increase in the plasma concentrations of several cytokines (CXCL8, CCL3, CCL4, and CXCL10) in the leprosy, T1LR and T2LR, and HHC groups. On the other hand, the levels of CCL2, CCL5, and CCL11 did not increase. Of note is that our study indicated that the CXCL8 concentration in plasma as a parameter had the highest accuracy and significance (AUC = 0.98, *p* = 0.0002) in classifying NI *vs*. HHC. Moreover, this chemokine was increased significantly at a magnitude as high as 68.5 times compared with NI. CCL3 was the single analyte with moderate accuracy and significance (AUC = 0.74, *p* = 0.0422) in classifying L(PB) *vs*. L(MB); it was increased 15.2× in L(MB) and 3.6× in L(PB) compared with NI.

CXCL10 is an important chemokine with the ability to recruit and activate specific subpopulations of leukocytes into sites of tissue damage and can act as a potent mediator of inflammation. CXCL10 also participates in cellular interactions, shaping and contributing to the development of immune response (36). This chemokine can be induced by IFN family molecules, leading to a robust cellular immune response, with intensive recruitment of CXCR3^+^ effector lymphocytes into skin lesions (37–39). Elevated levels of the chemokine CXCL10 were observed in PB and MB patients in relation to the group of individuals without a clinical history of leprosy (18, 34, 36). Moreover, it has been associated with neuritis of leprosy reactions and is also common in leprosy reactions (19, 40). The results of our study are in accordance with previously described findings, and our analysis of the fold change magnitude in plasma showed that CXCL10 was increased 2.8× in the L(PB), 3.8× in the L(MB), 4.9× in the T1RL, and 5.2× in the T2RL group compared with the NI healthy controls. It is a consensus among some authors that CXCL10 can be used as a prognostic biomarker for these diseases.

As is known, cytokines are immunological mediators essential in the course of leprosy, and they are associated with the different clinical forms of the disease. IFN-γ and TNF-α have been described as important cytokines in the elimination of the bacillus. They stimulate the expression of nitric oxide and induce the cellular immune response (7, 9, 11, 12). In addition, when the level of INF-γ is high, it may be involved in the process of inflammation of the nerves, causing neurites to be present in leprosy reactions (41). Our results showed that the IFN-γ levels in patients with leprosy increased 2.5× in the L(PB), 4.5× in the L(MB), 5.7× in the T1RL, and 9.7× in the T2RL group, as well as 7.6× in the HHC group, compared with the NI healthy controls (**Figure 1**). Another cytokine evaluated in this work was IL-9. Although IL-9 is known as a modulatory cytokine, Finiasz et al. (42), who carried out a study based on peripheral blood mononuclear cells from non-diseased donors in culture under stimulation of inactivated *M. leprae*, showed the pro-inflammatory role of IL-9. The authors observed that this cytokine could modulate the effects of IL-4, IL-10, and IL-13, acting in synergy with IFN-γ and IL-6. Previous immunohistochemical analysis of lesions from patients with leprosy showed that IL-9 was more expressed in lesions of tuberculoid leprosy compared with lepromatous leprosy (43). Statistical analysis of the DT algorithms showed IL-9 as an attribute with moderate accuracy and significance (AUC = 0.77, *p* = 0.0041) in classifying T1LR *vs.* T2LR, with 73% of T2RL showing high levels of this cytokine (>9 pg/ml) in the plasma. Moreover, the analysis of IL-9 followed by IL-6 and CXCL10 classified T1RL *vs.* T2RL with high accuracy (96%). Another modulatory cytokine evaluated in this work was IL-1Ra. This cytokine has been reported as important in leprosy as it was found in high concentrations in the lesions of patients with different clinical forms of leprosy (25). The authors suggested IL-1Ra as a possible biomarker that can be used in the diagnosis of the disease. This cytokine has anti-inflammatory functions, acting as an antagonist of the cytokines IL-1α and IL-1β, competing for the same receptor. In our work, the results also showed an increase in the plasma levels of IL-1Ra in patients with paucibacillary and multibacillary leprosy and in HHC. The cytokine IL-17 also has an important role in the immune system, playing an effective and pleomorphic role during the pro-inflammatory response of the disease and modulating macrophage activity by inducing the production of TNF, IL-6, and iNOS. IL-17 has been associated with inflammation that causes cell demyelination, leading to damage to the peripheral nerves and contributing to leprosy reactions (6, 44), which could remain in the lesions even after treatment (12, 37, 44, 45). Contrary to expectations, in this study, IL-17 showed only a slight increase, while IL-6 had a more pronounced increase.

Cell growth factors comprised another group of immune response mediators evaluated in this work. Cell growth factor molecules are directly related to the proliferation, differentiation, and cell migration of neutrophils and macrophages and the stimulation of T cells at the site of inflammation. In leprosy, there are only a few studies in the literature on the involvement of cell growth factors. Stefani et al. (19) mentioned that IL-7 and PDGF represent potential biomarkers of erythema nodosum leprosum (T2LR). Fiallo et al. (46) found that vascular endothelial growth factor (VEGF) and its receptor KDR are highly expressed in granuloma cells from patients with a reverse reaction. Fiallo et al. also reported that VEGF is not only relevant during hyperpermeability and the differentiation of mononuclear cells but also that this molecule is involved in the initiation of the reverse reaction, when dendritic cells are activated in response to antigenic stimulation. These molecules (PDGF and G-CSF) are also considered important in inflammatory processes as they have regulatory/effector properties that function at injury sites, promoting migration, differentiation, proliferation, and cell growth (46–53). The results of the present study showed that the PDGF and G-CSF molecules were increased in the plasma of patients with leprosy and their contacts. The increase in these factors in the plasma of patients with leprosy, as observed in our results, could, in some way, be related to the lesions both at the skin and at the neural level (51, 52).

Despite being able to extract a substantial amount of data from the samples provided, it is crucial to recognize that a limitation of the study was the reduced number of samples evaluated. Future studies are required to evaluate the immunological profiles of patients with different clinical forms of leprosy in order to expand our understanding of the role of cytokines in the course of infection and to validate the applicability of the selected immune mediators in clinical practice.

## Conclusion

The use of robust statistical tools such as the ROC curve and DT algorithms supported the potential use of plasma immune mediators as complementary laboratory biomarkers for the classification of patients with leprosy and leprosy reactions and for the differentiation of HHC from NI. Along this line, CXCL8, CCL3→IFN-γ, and IL-9→IL-6→CXCL10 were the immune mediators/axis with higher accuracy in classifying NI *vs.* HHC, L(MB) *vs.* L(PB), and T1RL *vs.* T2RL, respectively.

## Data Availability

The raw data supporting the conclusions of this article will be made available by the authors, without undue reservation.
